# Endogenous SLPI contributes to the regulation of inflammatory responses in peritoneal macrophages by modulating MMP-9 production

**DOI:** 10.3389/fimmu.2025.1563845

**Published:** 2025-05-27

**Authors:** Mariia Tyshchenko, Natalia Pocałuń, Patrycja Kwiecińska, Joanna Cichy, Mieszko M. Wilk, Ewa Oleszycka

**Affiliations:** ^1^ Department of Immunology, Faculty of Biochemistry, Biophysics, and Biotechnology, Jagiellonian University, Kraków, Poland; ^2^ Doctoral School of Exact and Natural Sciences, Jagiellonian University, Kraków, Poland

**Keywords:** SLPI, proteinase inhibitor, LPS, septic shock, inflammation, monocytes, peritoneal macrophages, MMP-9 (matrix metalloproteinase-9)

## Abstract

Secretory leukocyte protease inhibitor (SLPI) is described as a potent regulator of inflammation and tissue homeostasis with pleiotropic functions. It has been shown to inhibit pro-inflammatory responses in myeloid cells. However, its expression patterns and specific functions in different monocyte and macrophage populations remain poorly understood. Therefore, we investigated its expression patterns in murine tissue macrophage populations by analysis of publicly available datasets and flow cytometry. Among various tissues, peritoneal macrophages were identified as a major source of SLPI, suggesting the highest impact of this inhibitor on their physiological and pathophysiological functions. To elucidate the role of SLPI in the inflammatory response, SLPI-deficient mice were used. First, the response to LPS was compared in resident and thioglycolate-recruited peritoneal macrophages. Moreover, we evaluated the role of SLPI in an *in vivo* mouse model of LPS-induced septic shock. Results demonstrated that while the lack of SLPI did not affect pro-inflammatory cytokine production in activated resident macrophages, it regulated the production of matrix metalloproteinase-9 (MMP-9). Similar results were observed in thioglycolate-elicited and LPS-activated peritoneal macrophage populations, further highlighting the link between SLPI and MMP-9. Furthermore, *in vivo* LPS-induced changes in SLPI expression were evident among various myeloid populations, including monocytes. Loss of SLPI also influenced the frequency of blood monocyte populations in this model. Overall, these findings highlight a specific role for SLPI in regulating MMP-9 in response to LPS both *in vitro* and *in vivo* and suggest that SLPI might play a role in tissue remodeling orchestrated by macrophages.

## Introduction

Secretory leukocyte proteinase inhibitor (SLPI) is a potent regulator of inflammation and tissue homeostasis, mainly due to its antiproteolytic activity (1). It is detected in many tissues, including the respiratory and digestive tracts (2–4). It is secreted by epithelial cells, but also by immune cells (5). Furthermore, SLPI has been shown to be stored in granules of neutrophils, eosinophils, basophils, and mast cells (6–9). One of the main functions of SLPI is to inhibit serine proteases including neutrophil-derived elastase and cathepsin G and mast cell-derived chymase (10–12). In addition to its antiproteolytic activity, SLPI also plays other roles in tissue homeostasis. It has been reported that SLPI possesses anti-microbial properties and can inhibit bacterial and fungal growth (13, 14). Furthermore, SLPI has been shown to be an anti-inflammatory modulator of immunity. For instance, SLPI inhibits NF-κB pathway activation as it has been demonstrated that it can suppress degradation of inhibitory components IκBα and IκBβ of the complex and it can also compete with NF-κB p65 subunit to bind to promotor of proinflammatory genes (15, 16). Furthermore, SLPI plays a protective role in endotoxin shock and sepsis, as mice lacking SLPI develop more severe inflammation and increased mortality (17). SLPI also plays an important role in lung homeostasis. It was shown that airway microbiota composition influences SLPI levels in the respiratory system and can affect the development of allergic asthma inflammation (18). Additionally, mice deficient in SLPI develop severe chronic allergic inflammation (19). SLPI has also been described to modulate inflammatory responses during urinary tract *Escherichia coli* infection and *Pseudomonas aeruginosa* lung infection (20, 21).

Matrix metalloproteinases (MMPs) are enzymes that are necessary for degradation of extracellular matrix during physiological processes, but can also play a role in various diseases (22). MMP-9 also known as gelatinase-B digests elastin, collagen and gelatine (denatured collagen) and is involved in the remodeling of the extracellular matrix (ECM) (23). Importantly, while crucial for physiological processes, excessive MMP-9 activation is associated with many fibrosis-related diseases such as idiopathic pulmonary fibrosis, hepatic and renal fibrosis (24). Both SLPI and MMP-9 are involved in inflammation and the link between them was reported in the context of as cancer (25) and lung diseases (26, 27).

Overall, while SLPI is considered an important player in anti-inflammatory responses, its specific cellular and molecular targets are still elusive in specific diseases. Moreover, while the role of SLPI in macrophage activation is recognized (15–17, 28, 29), previous studies have not taken into account the heterogeneity of macrophages.

In this study, we investigated the expression pattern of SLPI in various macrophage populations. Subsequently, having established that resident peritoneal macrophages produce high levels of endogenous SLPI, we show that in these cells SLPI does not play a role in the secretion of pro-inflammatory cytokines but rather has a specific function in the regulation of MMP-9 production. Moreover, we describe that lipopolysaccharides (LPS)-induced endotoxin shock results in broad changes in SLPI expression across various myeloid cell populations and that SLPI influences frequency of monocytes. In general, this study reveals a novel function of SLPI in regulating the inflammatory response in local and systemic settings.

## Methods

### Animals

*Slpi* ^-/-^ mice were generated as previously described (30). C57BL/6 (referred as WT) and *Slpi* ^-/-^ mice were bred and housed in a specific pathogen-free animal facility at the Faculty of Biochemistry, Biophysics and Biotechnology of Jagiellonian University. The mice were matched in sex and age for experiments and were used between 8 and 12 weeks of age. All experiments were approved by the II Local Ethics Committee in Krakow (approval numbers 377/2021 and 142/2023).

### Reagents and antibodies

The following reagents were used: Alhydrogel (Cat. No. vac-alu-250) referred as “alum”, ultrapure LPS from *E. coli* serotype O55:B5 (Cat. No. Tlrl-pb5lps) for *in vivo* experiments, mouse TLR1–9 Agonist Kit (Cat. No. tlrl-kit1mw), nigericin (Cat. No. tlrl-nig) all from Invivogen (Toulouse, France); BD DIFCO fluid thioglycolate medium (Cat. No. 225650; BD, Argenta, Poznan, Poland); TMB substrate reagent set (Cat. No. 555214; BD, Warsaw, Poland), streptavidin PE (Cat. No. 554061; BD, Warsaw, Poland); LPS from *E. coli* serotype EH100 (Cat. No. ALX-581-010-L001; Enzo, Farmingdale, USA) for *in vitro* experiments; murine recombinant IFN-γ (Cat. No. 485-MI; R&D Systems, Bio-Techne, Warsaw, Poland); Peprotech murine recombinant IL-4 (Cat. No. 214-14), Peprotech murine recombinant M-CSF (Cat. No. 315-02), Gibco DMEM with high glucose (Cat. No. 11965092), penicillin-streptomycin (Cat. No. 15070063), Gibco heat-inactivated fetal bovine serum (Cat. No. 10500064), eBioscience Intracellular Fixation Buffer (Cat. No. 00-8222-49), eBioscience Foxp3/Transcription Factor Fixation/Permeabilization concentrate and diluent (Cat. No. 00-5521-00), eBioscience permeabilization buffer (10X) (Cat. No. 00-8333-56) all from Thermo Fisher Scientific (Life Technologies, Warsaw, Poland); inhibitors: JNK-IN-8 (Cat. No. HY-13319), SP600125 (Cat. No. HY-12041), PD98059 (Cat. No. HY-12028) and SB203580 (Cat. No. HY-10256) all from MedChemExpress (Sollentuna, Sweden); PBS (Cat. No. P04-36500; PAN-Biotech, Immuniq, Zory, Poland); sodium chloride injection solution (Cat. No. 3117301, Polpharma, Starogard Gdanski, Poland); BSA (Cat. No. ALB004; BioShop, Epro Science, Wladyslawowo, Poland); Tween-20 (Cat. No. P9416), saponin (Cat. No. 84510), gelatin type A from porcine skin (Cat. No. G-2625) all from Sigma-Aldrich (Merck Life Science, Poznan, Poland); Triton X-100 (Cat. No. 841810492; Poch SA, Gliwice, Poland); 2x Laemmli sample buffer (Cat. No. 1610737; Bio-Rad, Warsaw, Poland); WesternBright ECL HRP substrate (Cat. No. K-12045-D20, Advansta, San Jose, California, USA); paraformaldehyde (Cat. No. 43368.9M; VWR International, Gdansk, Poland); Zombie Aqua Fixable Viability Kit (Cat. No. 423102; Biolegend, Amsterdam, The Netherlands). The following antibodies were used: anti-CD3 PerCPCy5.5 (0.1 μg/100 μl; clone 17A2, Cat. No. 100218), anti-F4/80 Alexa Fluor 647 (0.25 μg/100 μl; clone BM8, Cat. No. 123122), anti-CD45 APCCy7 (0.2 μg/100 μl; clone 104; Cat. No. 109824), anti-Ly6C PE-Cy7 (0.005 μg/ml; clone HK1.4, Cat. No. 128017), anti-Ly6G FITC (0.25 μg/100 μl; clone 1A8, Cat. No. 127605) all from Biolegend (Amsterdam, The Netherlands); anti-CD11b BV650 (0.04 μg/100 μl; clone M1/70; Cat. No. 563402), anti-CD11c BV421 (0.2 μg/100 μl; clone HL3; Cat. No. 562782), anti-Ly6G BV711 (0.2 μg/100 μl; clone 1A8; Cat. No. 563979), anti-SiglecF PerCPCy5.5 (0.1 μg/100 μl; clone E50-2440; Cat. No. 565526) all from BD (Warsaw, Poland); anti-CD16/CD32 monoclonal antibodies (0.25 μg/100 μl; clone 93; Cat. No. 14-0161-85), eBioscience anti-c-kit FITC (0.25 μg/100 μl; clone 2B8; Cat. No. 11-1171-85), anti-CD11c PerCPCy5.5 (0.1 μg/100 μl; clone N418, Cat. No. 45-0114), anti-CD19 PerCPCy5.5 (0.1 μg/100 μl; clone 1D3, Cat. No. 45-0193-82), anti-MHC II Alexa Fluor 700 (0.02 μg/100 μl; clone M5/114.15.2, Cat. No. 56-5321-80), anti-SiglecF eFluor660 (0.04 μg/100 μl; clone 1RNM44N, Cat. No. 50-1702-82) all from Thermo Fisher Scientific (Life Technologies, Warsaw, Poland); anti-mouse SLPI – biotin (0.067 μg/100μl; Cat. No. BAF-1735), anti-mouse SLPI (2 μg/ml; Cat. No. AF1735), anti-mouse MMP9 (0.25 μg/ml; Cat. No. AF909-SP) all from R&D Systems (Bio-Techne, Warsaw, Poland); anti-β-actin (1:5000 dilution; clone AC15; Cat. No. A1978), rabbit anti-goat IgG antibody HRP conjugated (0.5 μg/ml; Cat. No. AP106P) all from Sigma-Aldrich (Merck Life Science, Poznan, Poland); StarBright™ Blue 520 goat anti-mouse IgG (1:2500 dilution; Cat. No. 12005867, Bio-Rad, Warsaw, Poland).

### *In vivo* models

Mice were injected intraperitoneally with 100 µg alum, 50 μg LPS or 1 ml of 4% thioglycolate. As controls PBS (for alum and LPS) and sodium chloride injection solution (for thioglycolate) were used. Mice were sacrificed after the time indicated in the figure legends.

### Cell culture

Bone marrow-derived macrophages (BMDMs) were generated as previously described (31). Briefly, bone marrow cells were isolated from the tibias and femurs of mice. Cells were grown in DMEM supplemented with 10% FBS, 50 units/ml of penicillin and 50 μg/ml of streptomycin (complete DMEM) and 20 ng/ml M-CSF. On day 5 or 6 non-adherent cells were removed, and macrophages were scraped and plated at a density of 1×10^6^/ml in complete DMEM with 20 ng/ml M-CSF. Cells were left overnight and stimulated as indicated in the figure legends.

Peritoneal macrophages were isolated from naïve mice or mice injected intraperitoneally with 1 ml of 4% thioglycolate. Cells were collected by washing the peritoneal cavity of mice with PBS and pelleted by centrifugation (400 × g, 5 min, 4˚C). Cells were plated onto flat bottom plates at a density of 2×10^6^/ml in complete DMEM. Macrophages were allowed to adhere for at least 2 h (37˚C, 5% CO_2_) and were washed twice with PBS to remove non-adherent cells. After adding fresh medium, peritoneal macrophages were stimulated as indicated in the figure legends.

For the proteome array kit and inhibitor treatment, peritoneal macrophages were MACS-sorted from peritoneal cells using Macrophage Isolation Kit (Peritoneum), mouse (Cat. No. 130-110-434; Miltenyi Biotec, BioLike, Wieliczka, Poland) according to manufacturer’s protocol. Isolated peritoneal macrophages were seeded at density 0.5×10^6^/ml in complete DMEM onto 24-well plate and stimulated as indicated in the figure legends.

### Flow cytometry staining

**Figure 1:**
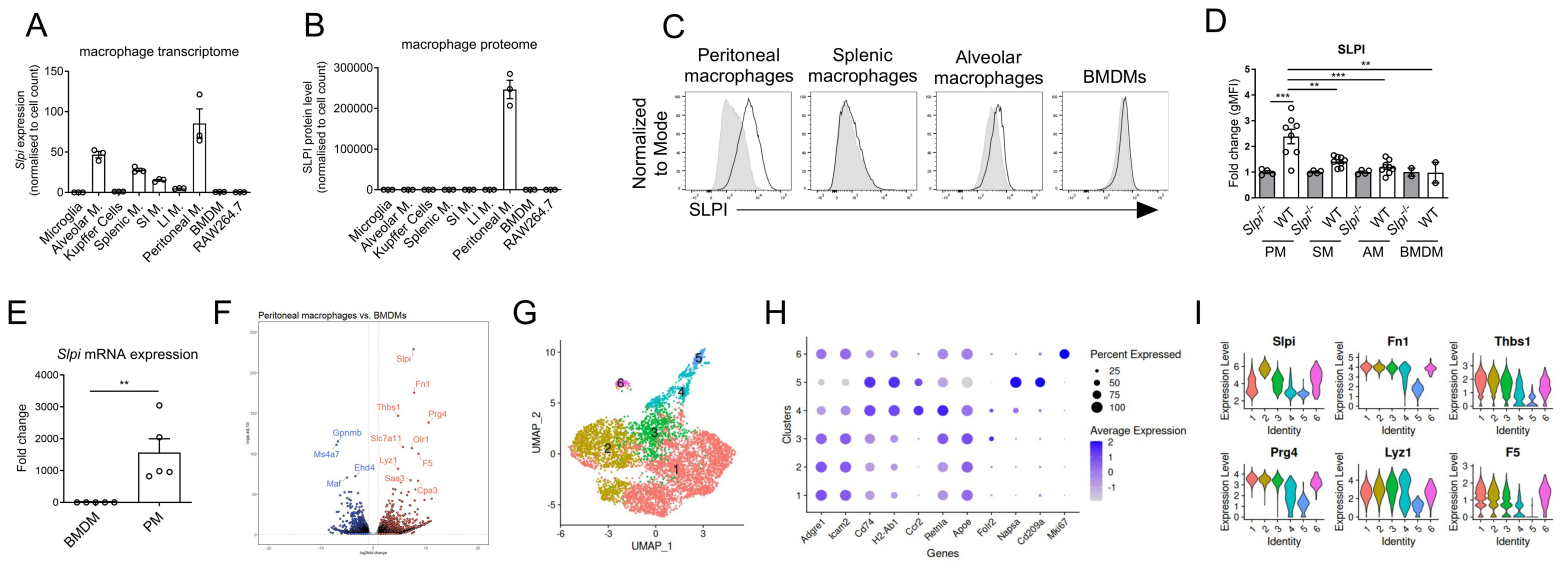
Heterogenous expression of SLPI in steady-state conditions in murine macrophages. Comparison of *Slpi* transcript **(A)** and SLPI protein level **(B)** in different macrophage populations. Data obtained from publicly available dataset. **(C)** Representative SLPI expression in freshly isolated peritoneal, splenic, alveolar macrophages and BMDMs from WT (black line) and *Slpi* ^-/-^ mice (shaded histogram). **(D)** Fold change of geometrical MFI was compared between WT and *Slpi* ^-/-^ macrophages as in **(C)** Data pooled from n=2-7. WT vs *Slpi* ^-/-^ **p < 0.01, ***p < 0.001 by one-way ANOVA, Tuckey *post hoc* test. **(E)** qRT-PCR analysis of *Slpi*. mRNA expression by freshly isolated BMDMs and peritoneal macrophages. Data pooled from n=5. WT BMDM vs WT peritoneal macrophages **p < 0.01 by unpaired two-tailed t-test. **(F)** Volcano plot of differentially expressed genes (log2 fold change > 1 and p-adj < 0.05) between BMDMs and peritoneal macrophages with top 10 upregulated (red) and downregulated (blue) genes highlighted on the plot. **(G)** UMAP of CD11b^+^ peritoneal cells. **(H)** Dot plot of 6 clusters from CD11b^+^ peritoneal cells. **(I)** Violin plots with expression level of *Slpi*, *Fn1*, *Thbs1*, *Prg4*, *Lyz1*, *F5* in 6 clusters from CD11b^+^ peritoneal cells.

Cells were washed with PBS, pelleted by centrifugation (400 × *g* for 5 min at 4°C) and stained with Zombie Aqua (dilution 1:1000 in PBS) for 30 min in the dark and on ice. After washing with FACS buffer (1% BSA in PBS), cells were incubated with 50 μl of FACS buffer mixed with anti-CD16/CD32 monoclonal Abs. Cells were then stained with the fluorochrome-labeled Abs for 20 min in the dark and on ice. Cells were washed twice and resuspended in 100 μl of FACS buffer and fixed using 100 μl Intracellular Fixation Buffer. Alternatively, for intracellular staining, cells were fixed with 200 μl Fixation/Permeabilization Buffer (prepared from Foxp3/Transcription Factor Fixation/Permeabilization Concentrate and Diluent). The cells were permeabilized and stained with 200 μl 1× Permeabilization Buffer prepared from 10× Permeabilization Buffer. Following staining, cells were washed twice using 1× Permeabilization Buffer and resuspended in 200 μl of FACS buffer. Alternatively, cells were fixed with 4% PFA and permeabilized with 1% saponin (data for **Figure 1**). All compensations were set up using UltraComp eBeads (Cat. No. 01-2222-42; Invitrogen, Thermo Fisher Scientific, Life Technologies, Warsaw, Poland), or if not applicable, cells were stained with a dye. Samples were acquired on BD LSR II using FACSDiva (BD Biosciences), and the data were analyzed using FlowJo software version 10.10.0 (BD).

### Western blotting

Cells were cultured in 12-well plates (Cat. No. 83.3921.300; Sarstedt, Warsaw, Poland) (1 × 10^6^ cells/ml; 1 ml) and stimulated as described in the figure legends. For whole cell lysate analysis, cells were lysed with 2 × SDS-PAGE sample buffer and heated to 95°C for 5 min. For supernatant analysis, supernatants were diluted 1:1 using 2 × SDS-PAGE sample buffer and heated to 95°C for 5 min. The samples were resolved by SDS-PAGE, transferred to PVDF membranes, and analyzed by immunoblot with appropriate Abs. Immunoreactivity was visualized by ECL or fluorescent antibodies. Densitometry analysis was performed using ImageJ software version 1.54i.

### Gelatin zymography

Supernatants from cell culture were diluted in non-reducing loading dye and resolved by SDS-PAGE in a 7% acrylamide gel containing 0.67 mg/ml gelatin. After protein separation, the gels were washed twice for 30 minutes in wash buffer (2.5% Triton X-100 in 50 mM Tris, pH 7.7) and incubated in activation buffer (50 mM Tris, pH 7.7, 5 mM CaCl_2_, 1 μM ZnCl_2_) for 16 hours at 37°C. Gels were stained with 0.5% Brilliant Blue R-250 (Cat. No. 6104-59-2; Sigma-Aldrich, Merck Life Science, Poznan, Poland) in 40% methanol and 10% acetic acid.

### RT-qPCR

Total RNA was extracted from cells using Fenozol Plus (Cat. No. 203-100P; A&A Biotechnology, Gdansk, Poland) with Total RNA Zol-out kit (Cat. No. 030-100; A&A Biotechnology, Gdansk, Poland) according to manufacturer’s protocol. cDNA was generated from up to 400 ng RNA using NxGen M-MuLV Reverse Transcriptase (Cat. No. 30222-1; LGC Biosearch Technologies, Hoddesdon, UK) with random hexamers (Cat. No. N8080127, Invitrogen, Thermo Fisher Scientific, Life Technologies, Warsaw, Poland), oligo(dT) (synthetized by Genomed, Warsaw, Poland) and dNTPs (Cat. No. U1240; Promega, Walldorf, Germany). Next, real-time RT PCR analyses were performed using RT HS-PCR Mix SYBR (Cat. No. 2017-100HS; A&A Biotechnology, Gdansk, Poland) according to the manufacturer’s instructions. The abundance of each mRNA was normalized relative to PCR of the housekeeping gene hypoxanthine-guanine phosphoribosyltransferase (*Hprt*) from the corresponding sample (Ct_Gene_ – Ct_HPRT_ = ΔCt). Furthermore, ΔCt from control samples were averaged and were subtracted from the ΔCt of each sample (ΔCt_1_ − ΔCt_ctrl_ mean = ΔΔCt_1_). Fold induction was calculated as 2^(−ΔΔCt)^. The following primers were used: mouse *Hprt1* (NM_013556.2), forward AGGGATTTGAATCACGTTTG and reverse TTTACTGGCAACATCAACAG; mouse *Slpi* (NM_011414.4, NM_001412601.1), forward GTCCTGCGGCCTTTTACCTT and reverse TACGGCATTGTGGCTTCTCA. Controls for the PCR product were conducted to verify product specificity, including negative control water sample and melting curve analysis.

### Measurement of cytokine levels by ELISA

Concentrations of IL-1α (Cat. No. DY400-05), IL-1β (Cat. No. DY401), TNF (Cat. No. DY410), IL-6 (Cat. No. DY406), SLPI (Cat. No. DY1735-05), MMP-9 (Cat. No. DY6718) were measured using kits from R&D Systems (Bio-Techne, Warsaw, Poland) according to manufacturer’s instructions.

### Proteome profiler array

Proteome Profiler Mouse XL Cytokine Array (Cat. No. ARY028, R&D Systems, Bio-Techne, Warsaw, Poland) was used according to manufacturer’s instruction to analyze secretome of peritoneal macrophages.

### Analysis of publicly available expression data

Proteomic and transcriptomic macrophage data were obtained from publication and corresponding datasets (32). Primary macrophage populations (lung: alveolar macrophages, brain: microglia, liver: Kupffer cells, spleen: red pulp macrophages, small intestine macrophages, large intestine macrophages, peritoneal macrophages) were isolated by FACS-sorting from naïve mice. Bone marrow derived macrophages (BMDMs) and cell line RAW264.7 were cultured. Individual macrophage populations were analyzed by mass spectrometry and bulk RNA-seq. Processed data presented in this manuscript is available at the server (http://macrophage.mouseprotein.cn/). RNA-seq data for the differences between untreated BMDMs and peritoneal macrophage populations were obtained from GEO GSE179504 (33). Briefly, raw counts from triplicate samples representing BMDMs and peritoneal macrophages were selected. Analysis was performed using Differential Gene Expression (DESeq2) (34) in R. Raw counts for scRNAseq analysis of FACS-sorted CD11b^+^ peritoneal cells were obtained from GSE139999 (35). Briefly, low quality cells were filtered out using thresholds such as number of genes per cell, number of reads per cell, percentage of mitochondrial genes per cell. Data integration and clustering was done using CCA in Seurat with default parameters and 12 principal components. Clusters were merged manually to show cell populations with unique markers.

### Statistical analysis

The statistics were analyzed using GraphPad Prism 9 (GraphPad Software). The unpaired two-tailed t test was used when two groups were compared; when multiple groups were compared, one-way ANOVA were used.

## Results

### Endogenous SLPI is highly expressed in murine peritoneal macrophages

SLPI expression has been studied in the context of several myeloid cells (5, 6, 28, 36, 37), however analysis of its expression among diverse populations of tissue macrophages was never explored. Thus, we took advantage of the existing macrophage atlas (macrophage.mouseprotein.cn), which provides integrated transcriptomic and proteomic analysis of FACS-sorted macrophages from various murine tissues (32). Among selected resident macrophages from brain, lungs, liver, spleen, intestine, peritoneum and also bone marrow-derived macrophages (BMDMs) and murine cell line RAW246.7, the highest *Slpi* expression was detected in peritoneal macrophages (**Figure 1A**). The *Slpi* transcript was also detected in lung alveolar macrophages, red pulp splenic macrophages, and small intestine macrophages. However, from proteomic dataset, only peritoneal macrophages expressed SLPI protein, while in other macrophage populations it was not detected (**Figure 1B**). To confirm these results, SLPI expression was analyzed in selected murine macrophage populations by flow cytometry including large peritoneal macrophages, splenic macrophages, alveolar macrophages and BMDMs. *Slpi* ^-/-^ cells were used as negative staining controls. Similarly to database results, WT large peritoneal macrophages expressed the highest levels of SLPI and these levels were significantly higher than those in BMDMs, splenic or alveolar macrophages (**Figure 1C, D**).

Next, we confirmed that peritoneal macrophages highly express *Slpi* mRNA when compared to BMDMs (**Figure 1E**). We also analyzed differential gene expression between BMDMs and peritoneal macrophages using RNA-Seq data set (33). Interestingly, *Slpi* is among the top highly expressed genes in peritoneal macrophages when compared to BMDMs (**Figure 1F**).

Peritoneal macrophages comprise distinct populations that differ in origin and functions with two major population: large and small peritoneal macrophages (35, 38–41). To better understand *Slpi* expression in heterogenous peritoneal macrophages, dataset of CD11b^+^ peritoneal cells was analyzed (35). Within CD11b^+^ cells, there were 5 clusters of macrophages (1, 2, 3, 4 and 6) which expressed *Adgre1*, *Icam2* and cluster number 5 which represents dendritic cells expressing *Napsa*, *Cd209a* and *H2-Ab1* (**Figure 1G, H**). Peritoneal macrophages were divided into 5 clusters depending on their expression of *Cd74*, *H2-Ab1*, *Retnla*, *Apoe*, *Folr2* and *Mki67*. Next, we analyzed expression of genes which were differentially expressed by peritoneal macrophages such as *Slpi*, *Fn1*, *Prg4*, *Lyz1*, *F5* and *Saa3*. Within macrophage clusters, *Slpi* was highly expressed by cluster 2, while its expression was lower in other clusters. Interestingly, *Fn1*, *Thbs1*, *Prg4*, *Lyz1* and *F5* were expressed at similar levels in clusters 1, 2 and 3, while clusters 4 express these genes at lower level. Overall, these results further support our findings that SLPI is constantly expressed by peritoneal macrophages. Flow cytometry analysis show that peritoneal macrophages comprise two populations: large peritoneal macrophages (LPMs) and small peritoneal macrophages (SPMs) (**Supplementary Figure S1A**) (41) and we investigated whether they differ in SLPI expression. Interestingly, both LPM and SPM express SLPI (**Supplementary Figure S1B**).

### Endogenous SLPI has the selective effect on the LPS response in peritoneal macrophages

**Figure 2:**
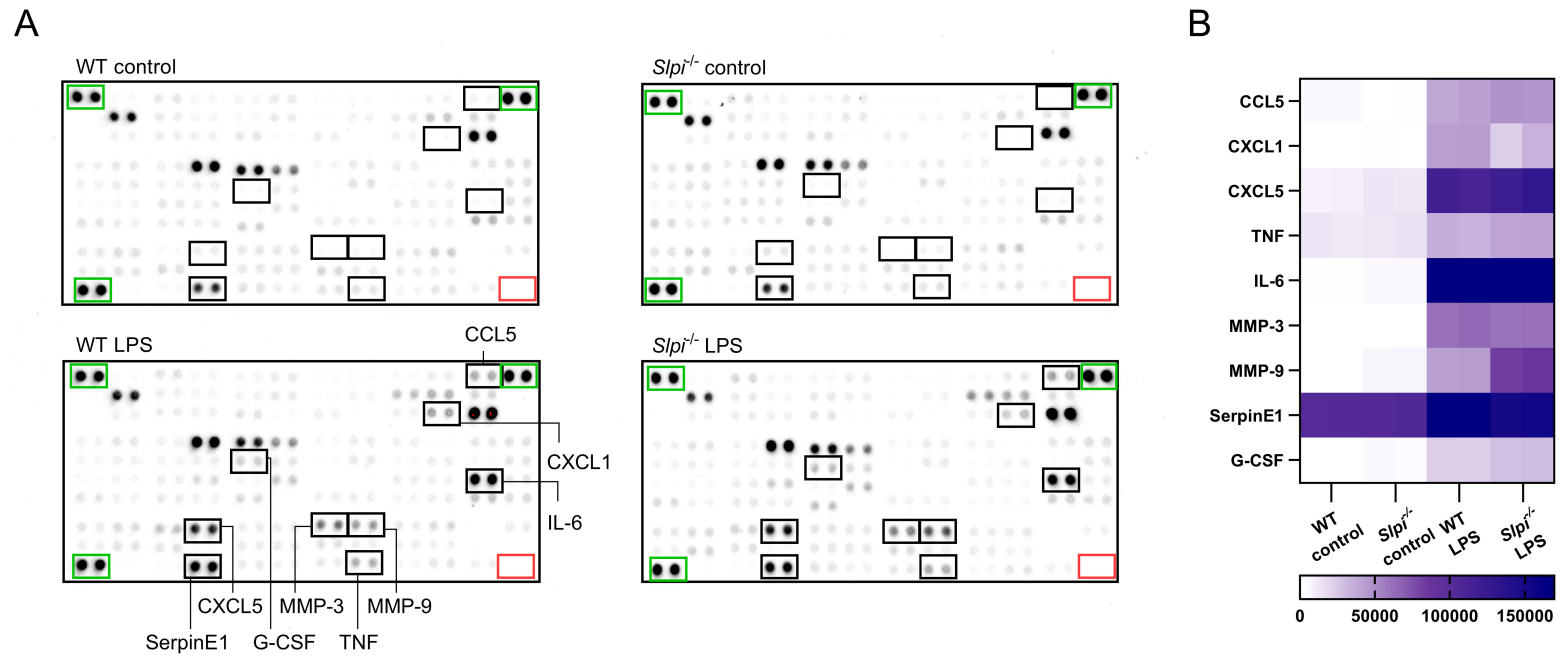
One sample analysis of cytokine proteome array. **(A)** The array images for supernatants of WT and *Slpi* ^-/-^ resident peritoneal macrophages stimulated with LPS (100 ng/ml) for 48h. Negative duplicate control spots are marked by red rectangle. Positive signal reference spots are marked by green rectangle. **(B)** Heatmap of relative levels (pixel density) of selected cytokines secreted by peritoneal macrophages.

Having confirmed, that SLPI is highly expressed by peritoneal macrophages in steady state, we next investigated how constitutive endogenous SLPI impacts inflammatory response in macrophages. To obtain highly pure peritoneal macrophages, we utilized MACS isolation kit, which gave >95% purity with >95% of LPMs (**Supplementary Figure S1C**). While isolated cells comprise almost pure population of large peritoneal macrophages, we cannot exclude the effect of small peritoneal macrophages contamination, thus we called isolated cells resident peritoneal macrophages. We selected LPS for macrophage stimulation as this TLR4 ligand is routinely used in many studies deciphering macrophage biology (42, 43). Different doses of LPS were analyzed and as expected increasing concentration of LPS induced higher SLPI secretion in WT peritoneal macrophages (**Supplementary Figure S2**). To undertake an unbiased approach, we compared the secretome of WT and *Slpi* ^-/-^ peritoneal macrophages stimulated with control or 100 ng/ml LPS using the proteome profiler array kit. This analysis allowed us to assess 114 cytokines, chemokines, growth factors and other mediators in the samples. We evaluated supernatants from cells incubated with control or LPS for 48 h to allow for mediator accumulation. However, since this approach analyses only one time point, it is possible that some mediators are lost due to their short half-lives. Despite this limitation, as expected LPS treatment induced a significant up-regulation of selected proteins, including, chemokines (CCL5, CXCL1, CXCL5), cytokines (IL-6 and TNF), proteinase inhibitors (SerpinE1), growth factor (G-CSF) and proteinases (MMP-3 and MMP-9) (**Figure 2A**). However, there were no broad differences in secretion of these various mediators between WT and *Slpi^-/-^* peritoneal macrophages (**Figure 2A**). In contrast to previous reports showing the role of SLPI in NF-κB signaling pathway, proinflammatory cytokines IL-6 and TNF were secreted to the same extend in WT and *Slpi* ^-/-^ peritoneal macrophages. Additionally, secretion of proinflammatory chemokines CCL5 and CXCL1 and other mediators of inflammation were also comparable in these cells. Interestingly, in contrast to these mediators, we found that *Slpi* ^-/-^ peritoneal macrophages secreted approximately two-fold higher levels of MMP-9 than WT cells (**Figure 2B**).

### *Slpi* ^-/-^ resident peritoneal macrophages exhibit increased MMP-9 secretion

**Figure 3:**
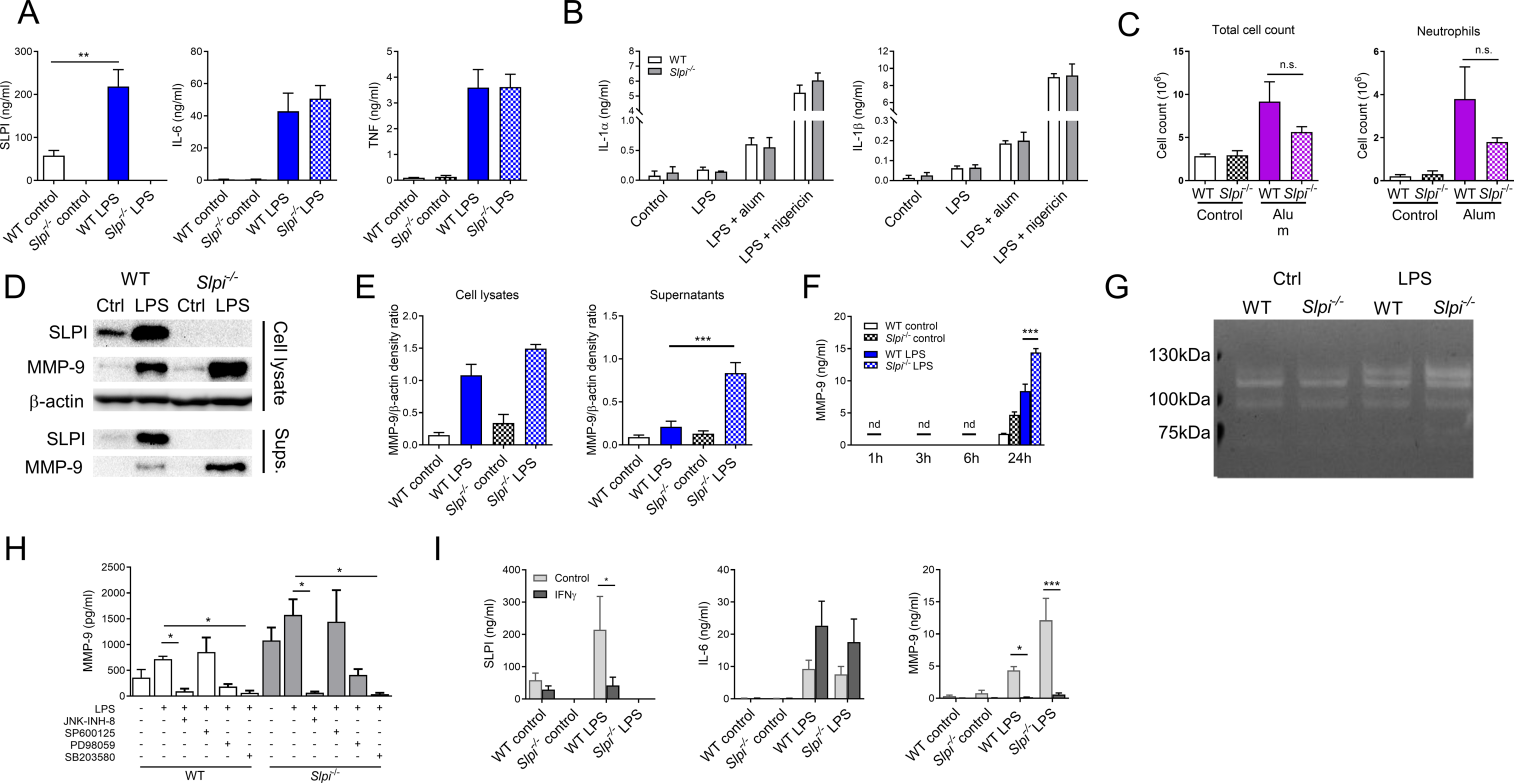
SLPI regulates MMP-9 secretion in resident peritoneal macrophages. **(A)** SLPI, IL-6 and TNF in supernatants of WT and *Slpi^-/-^* resident peritoneal macrophages incubated with LPS (100 ng/ml) for 24h.WT control vs WT LPS ** p< 0.01 by one-way ANOVA, Tuckey *post hoc* test. **(B)** IL-1α and IL-1β in supernatants of WT and *Slpi^-/-^* peritoneal macrophages incubated with LPS (100 ng/ml) for 3 h followed by addition of alum (100 µg/ml) or nigericin (10 µM). **(C)** Total cell and neutrophil count in WT and *Slpi^-/-^* mice injected i.p. with PBS or 100 μg alum for 24h. **(D)** Representative immunoblots of MMP-9 and SLPI in supernatants and lysates of WT and *Slpi^-/-^* peritoneal macrophages incubated with LPS (100 ng/ml) for 24h.Samples were resolved by SDS-PAGE and probed by Western blotting. β-actin was used as loading control. **(E)** Densitometry analysis of **(D)** WT LPS vs *Slpi* ^-/-^ LPS ***p < 0.001 by one-way ANOVA, Tukey *post hoc* test. **(F)** MMP-9 in supernatants of WT and *Slpi^-/-^* resident peritoneal macrophages incubated with LPS (100 ng/ml) for indicated times. WT LPS vs *Slpi* ^-/-^ LPS ***p < 0.001 by one-way ANOVA, Tukey *post hoc* test. **(G)** Representative zymography of equal volume of supernatants obtained from WT and *Slpi^-/-^* peritoneal macrophages incubated with LPS (100 ng/ml) for 24h.Representative of four separate experiments. **(H)** MMP-9 in supernatants of WT and *Slpi^-/-^* resident peritoneal macrophages incubated with DMSO or inhibitors: JNK-INH-8 (10 μM), SP600125 (20 μM), PD98059 (20 μM) or SB203580 (10 μM) for 1h followed by LPS treatment (100 ng/ml) for 24h.LPS vs LPS + inhibitor *p<0.05 by one-way ANOVA, Tukey *post hoc* test. **(I)** SLPI, IL-6 and MMP-9 in supernatants of WT and *Slpi^-/-^* resident peritoneal macrophages incubated with LPS (100 ng/ml) and IFN-γ (50 ng/ml) for 24h.Control vs IFN−γ *p<0.05, ***p<0.001 by multiple unpaired t-test. **(A, B, F, H, I)** Data are presented as the mean of three **(A, B, F)** or four **(H, I)** independent experiments. Error bars show means ± SEM. **(C)** Data represent 5 to 6 mice per experimental group pooled from two independent experiments. Error bars show means ± SEM.

To confirm the results obtained from single measurements on protein array membranes, we analyzed cytokine production by control and LPS-stimulated peritoneal macrophages. Consistent with flow cytometry and proteomic data, we found that peritoneal macrophages produce high levels of SLPI in the steady state, which is further enhanced during 100 ng/ml LPS treatment (**Figure 3A**). Next, in accordance with the results of the proteome array, we did not detect difference in the release of IL-6 and TNF between WT and *Slpi* ^-/-^ peritoneal macrophages (**Figure 3A**). Recently, it has been shown that the addition of exogenous SLPI regulates IL-1 production in monocytes (44). Thus, we investigated whether endogenous expression of SLPI has an impact on IL-1 secretion in primary macrophages. WT and *Slpi* ^-/-^ peritoneal macrophages were stimulated with 100 ng/ml LPS followed by treatment with NLRP3 inflammasome activators, 100 μg/ml alum or 10 μM nigericin (45, 46). Interestingly, we did not detect any difference in IL-1α or IL-1β release in WT and *Slpi* ^-/-^ peritoneal macrophages (**Figure 3B**). However, there are multiple mechanisms of IL-1 activation (47, 48). For instance, both IL-1 cytokines can be cleaved by various proteases leading to inflammasome-independent IL-1 processing (31, 47). Therefore, we analyzed whether SLPI regulates IL-1 functionality *in vivo*. 24 h after alum injection, there was a cell influx in both WT and *Slpi* ^-/-^ mice and lack of SLPI resulted in a slight, but not significant reduction of neutrophil recruitment into the site of injection (**Figure 3C**). These results suggest that IL-1 activation and release at least in these models are independent of SLPI.

Having established that SLPI does not modulate release of selected cytokines, we investigated its role in MMP-9 secretion. To confirm the results from protein array membranes, cell lysates and supernatant from WT and *Slpi* ^-/-^ peritoneal macrophages were analyzed for SLPI and MMP-9 production. As expected, LPS stimulation led to up-regulation and secretion of both SLPI and MMP-9 by WT peritoneal macrophages. In contrast, *Slpi* ^-/-^ cells did not produce any SLPI, while MMP-9 secretion increased significantly (**Figure 3D, E**, **Supplementary Figure S3**). Furthermore, we analyzed various time points following LPS treatment and confirmed that peritoneal macrophages release MMP-9 at 24 h with significant increase in case of *Slpi* ^-/-^ cells (**Figure 3F**). Next, we examined protease activity of MMP-9 by gelatin zymography and we confirmed that there is an increase in MMP-9 secretion by LPS-treated *Slpi^-/-^* peritoneal macrophages (**Figure 3G**, **Supplementary Figure S3**). The increase of MMP-9 of the size approximately 120 kDa might correspond to MMP-9 complex (49).

MMP-9 is secreted by macrophages during inflammation resolution as the mediator of ECM remodeling and tissue regeneration (50). As macrophages can respond to various stimuli, we were interested to investigate whether addition of anti-inflammatory cytokines would impact on MMP-9 production by macrophages. WT and *Slpi^-/-^* peritoneal macrophages were stimulated with IL-4 and similarly to LPS stimulation, deficiency in SLPI resulted in enhanced MMP-9 secretion (**Supplementary Figure S4**).

It has been shown that SLPI interacts with scaffold protein c-Jun N-terminal kinase-interacting protein 3 (JIP3) which is crucial for c-Jun N-terminal kinase-1 (JNK-1) activation (9). Moreover, MMP9 expression is regulated by mitogen-activated protein kinases (MAPK) signaling (51). Thus, we investigated whether inhibition of MAPK pathway would affect MMP-9 secretion in WT and *Slpi* ^-/-^ peritoneal macrophages using JNK (JNK-IN-8 and SP600125), ERK1/2 (PD98059) or p38 (SB203580) inhibitors (51, 52). Interestingly, MMP-9 secretion was inhibited significantly by JNK-IN-8 and SB203580 in LPS-stimulated cells, but not by SP600125 or only partly by PD98059 (**Figure 3H**). The difference between two JNK inhibitors might be due to the higher selectivity of JNK-IN-8 and off-target effects of SP600125 (53–55). Nevertheless, these results suggest that all investigated downstream pathways take part in LPS-induced MMP-9 secretion regardless of SLPI expression.

Macrophages sense their tissue environment, and pathogens (or PAMPs) induce their activation. Additionally, cytokines and interferons produced by other cells can also influence their pro-inflammatory responses. For instance, IFN-γ sensing by macrophages modifies their metabolism and transcriptional response to LPS (56). Interestingly, while IFN-γ upregulates proinflammatory cytokines, it downregulates SLPI and MMP-9 expression in activated macrophages (36, 57). Thus, we investigated whether IFN-γ has an effect on SLPI secretion and subsequently on MMP-9. As expected, IFN- γ upregulated IL-6 production by LPS-stimulated peritoneal macrophages, while SLPI and MMP-9 secretion decreased (**Figure 3H**). However, there was no difference between WT and *Slpi* ^-/-^ macrophages, which shows that the effect of IFN-γ on MMP-9 is independent of SLPI (**Figure 3I**).

### *Slpi* ^-/-^ recruited peritoneal macrophages display increased IL-6 and MMP-9 secretion

**Figure 4:**
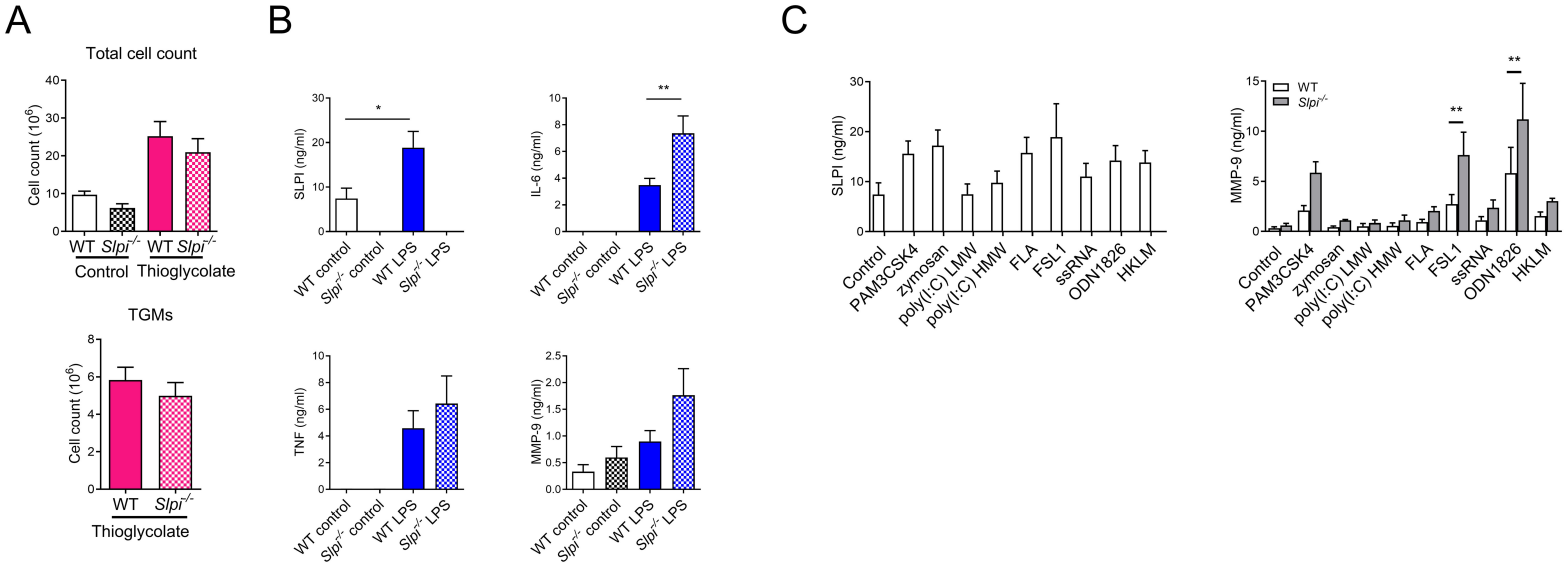
SLPI regulates proinflammatory responses in recruited peritoneal macrophages. **(A)** Total cell and thioglycolate-elicited macrophages (TGMs) count in WT and *Slpi* ^-/-^ mice injected i.p. with saline or 4% thioglycolate (1 ml) for 96h. **(B)** SLPI, IL-6, TNF and MMP-9 in supernatants of WT and *Slpi* ^-/-^ TGMs incubated with LPS (100 ng/ml) for 24h.WT control vs WT LPS *p<0.05; WT LPS vs *Slpi* ^-/-^ LPS **p<0.01 by one-way ANOVA, Tukey *post hoc* test. **(C)** SLPI and MMP-9 in supernatants of WT and *Slpi^-/-^* TGMs incubated with various TLR ligands (PAM3CSK4, 10 ng/ml; zymosan, 1 µg/ml; HKLM, 10^7^ cells; poly(I:C) HMW, 10 µg/ml; poly(I:C) LMW, 10 µg/ml; FLA, 1 µg/ml; FSL-1, 10 ng/ml; ssRNA, 2 µg/ml; ODN1826, 2 µg/ml) for 24h.WT TLR ligand vs *Slpi* ^-/-^ TLR ligand, ** p<0.01 by multiple unpaired t-test. **(A)** Data represent 4 to 6 mice per experimental group pooled from three independent experiments. Error bars show means ± SEM. **(B, C)** Data are presented as the mean of four independent experiments. Error bars show means ± SEM.

Next, we investigated the role of SLPI in thioglycolate-elicited macrophages (TGMs) as these macrophages are routinely used as a model to study inflammation. We hypothesized that there might be a difference between resident and recruited peritoneal macrophages as they represent different lineages (41). First, we analyzed whether SLPI would have an impact on thioglycolate-induced cell recruitment. However, there was no difference in both total cell number and macrophage cell count (**Figure 4A**). Next, we analyzed response of isolated WT and *Slpi* ^-/-^ TGMs to 100 ng/ml LPS stimulation. Similarly to resident peritoneal macrophages, WT TGMs secreted SLPI spontaneously in control treatment and increased levels after LPS stimulation (**Figure 4B**). Furthermore, we assessed the role of SLPI in the production of selected pro-inflammatory mediators in TGMs in response to LPS. In contrast to results in resident peritoneal macrophages, *Slpi* ^-/-^ TGMs released significantly more IL-6 following LPS, while MMP-9 was increased but not significantly when compared to WT cells (**Figure 4B**). Moreover, *Slpi* ^-/-^ TGMs did not upregulate TNF secretion (**Figure 4B**). Next, as macrophages respond to various pathogen-associated molecular patterns, we analyzed whether other TLR ligands might have more profound effect on MMP-9 production in TGMs. As expected TLR activation induced SLPI upregulation in WT TGMs (**Figure 4C**). Interestingly, FSL-1 (TLR2/6 ligand) and ODN1826 (TLR9 ligand) induced the highest MMP-9 production in TGMs which was further significantly increased in *Slpi* ^-/-^ TGMs (**Figure 4C**). In general, these results show that SLPI plays a diverse role in macrophage activation of different origin.

### SLPI influences the production of MMP-9 in peritoneal macrophages during the inflammatory response to LPS *in vivo*

**Figure 5:**
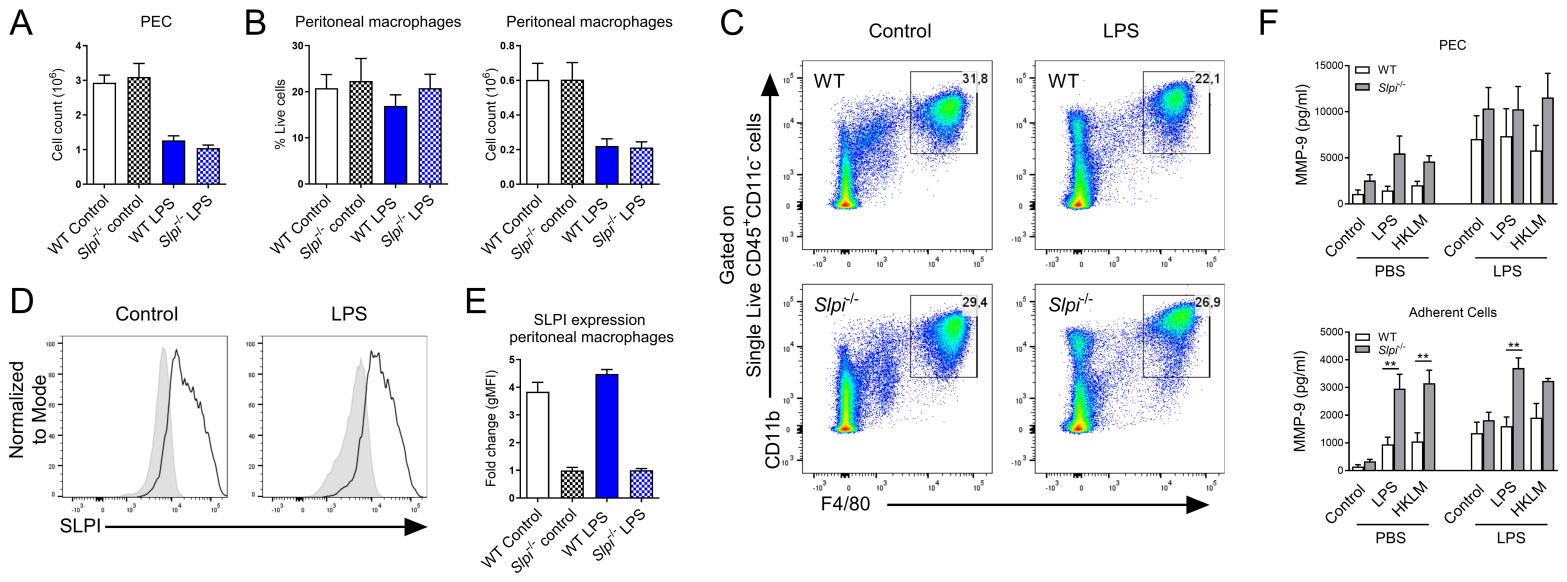
The impact of SLPI on local response to LPS injection. **(A)** Total PEC number isolated from WT and *Slpi^-/-^* mice injected with PBS or LPS for 24h. **(B)** Peritoneal macrophage percentage and total number as in **(A)**. **(C)** Representative gating for resident macrophages in WT and *Slpi^-/-^* mice injected with PBS or LPS for 24h.**(D)** SLPI expression in resident peritoneal macrophages isolated from WT (black line) and *Slpi* ^-/-^ mice (shaded histogram) injected with PBS or LPS for 24h.**(E)** Fold change of geometrical MFI was compared between WT and *Slpi* ^-/-^ macrophages as in **(D)**. **(F)** MMP-9 in supernatants of WT and *Slpi* ^-/-^ total PEC or peritoneal adherent cells (macrophages) isolated from PBS or LPS injected mice and stimulated with LPS (100ng/ml) or HKLM (10^7^ cells) for 24h.WT vs *Slpi* ^-/-^ **p<0.01 by multiple unpaired t-test. **(A, B, E)** Data represent 5 to 8 mice per experimental group pooled from three independent experiments. Error bars show means ± SEM. **(F)** Data represent 3 to 5 mice per experimental group pooled from two independent experiments. Error bars show means ± SEM.

Having established the role of SLPI in *in vitro* macrophage activation, we next assessed whether SLPI impacts inflammatory response during the *in vivo* inflammation. Peritoneal macrophages play crucial role in homeostasis and inflammatory responses (38) including sepsis (39, 58). As it has been shown that SLPI impacts septic shock and lethality in mice (17), we selected *in vivo* LPS-induced inflammation model. WT and *Slpi* ^-/-^ mice were injected with PBS or LPS intraperitoneally for 24 h and peritoneal exudate cells (PEC) were collected. Interestingly, the injection of LPS induced a significant decrease in total cell count at the site of injection in both WT and *Slpi* ^-/-^ mice at indicated time-point (**Figure 5A**). When comparing specific populations, peritoneal macrophages were present in comparable percentages, but their total count was decreased in both mouse strains after LPS treatment (**Figure 5B, C**). Next, we determined that SLPI was expressed in comparable levels by resident macrophages in the peritoneal cavity in PBS and LPS groups in WT mice (**Figure 5D, E**). In order to assess whether SLPI affected MMP-9 also during inflammatory settings, total peritoneal cells were stimulated with medium, TLR2 ligand, heat killed *Listeria monocytogenes* (HKLM) or TLR4 ligand, LPS and supernatants were analyzed for MMP-9. As expected, *Slpi* ^-/-^ total peritoneal cells secreted higher levels of MMP-9 compared to WT cells, and this secretion was further increased by LPS treatment *in vivo*. Moreover, *Slpi* ^-/-^ adherent cells, which represent enriched peritoneal macrophages, also secreted increased levels of MMP-9 (**Figure 5F**). Overall, these results confirm that SLPI influences MMP-9 production in various peritoneal macrophage populations during homeostasis and inflammatory settings.

### The expression of SLPI is modulated in monocytes and affects their frequency during the *in vivo* response to LPS

**Figure 6:**
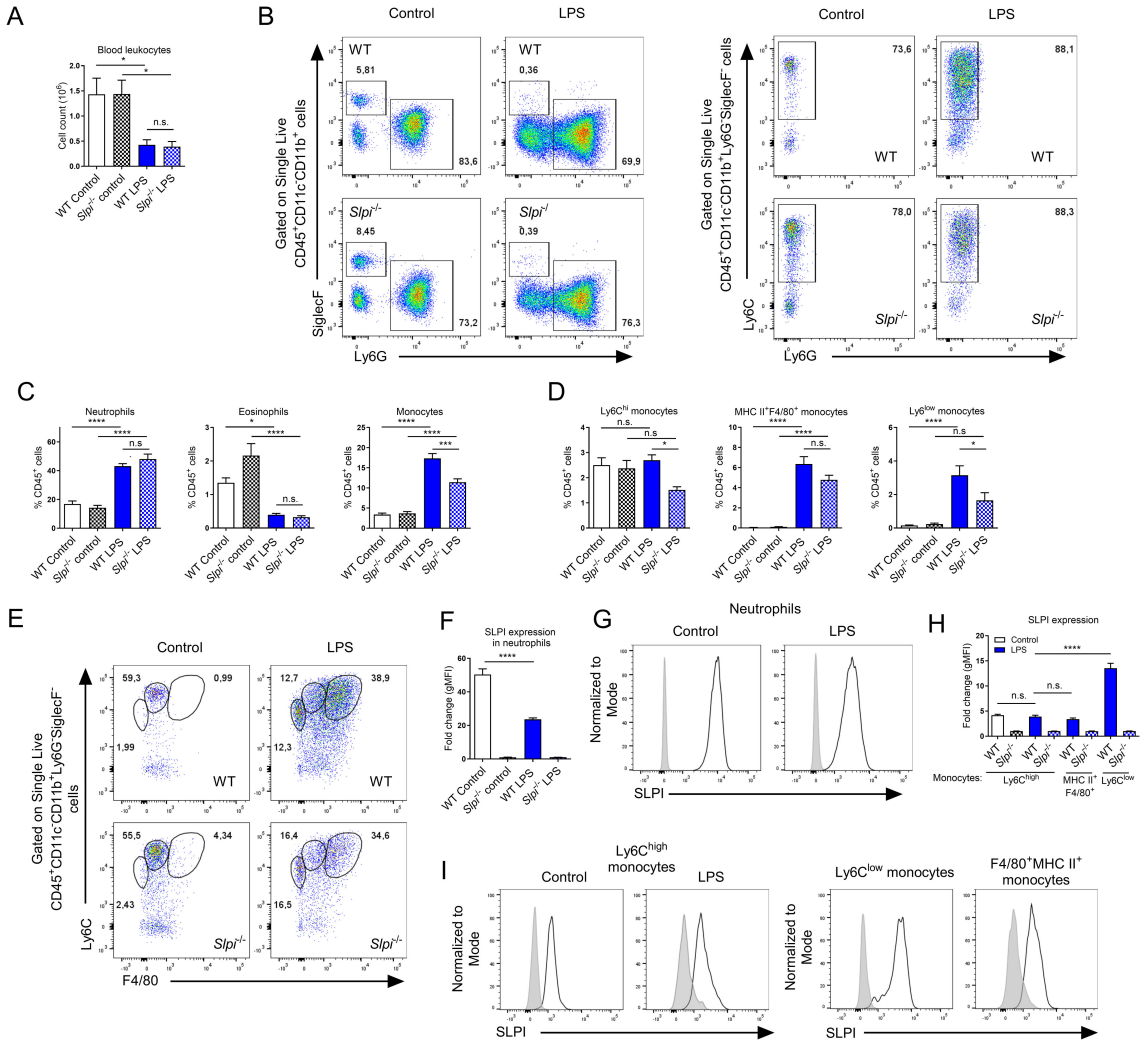
LPS induces changes in expression pattern of SLPI in selected immune cell populations *in vivo*. **(A)** Total blood CD45^+^ leukocyte number isolated from WT and *Slpi^-/-^* mice injected with PBS or LPS for 24h. **(B)** Representative gating for neutrophils (CD11b^+^Ly6G^+^SiglecF^-^), eosinophils (CD11b^+^Ly6G^-^SiglecF^+^) and monocytes (CD11b^+^Ly6C^+^Ly6G^-^) as in **(A)**. **(C)** Percentage of neutrophils, eosinophils and monocytes isolated as in **(A)**. **(D)** Percentage of Ly6C^hi^, MHC II^+^F4/80^+^ and Ly6C^low^ monocytes isolated as in **(A)**. **(E)** Representative gating of Ly6C^hi^ (CD11b^+^Ly6C^high^), MHC II^+^F4/80^+^ and Ly6C^low^ monocytes isolated as in **(A)**. **(F, G)** SLPI expression in blood neutrophils isolated from WT (black line) and *Slpi* ^-/-^ mice (shaded histogram) injected with PBS or LPS for 24h.Data represented as mean ± SEM of fold change in geometrical MFI values (WT vs *Slpi* ^-/-^). **(H)** SLPI expression in blood monocytes represented as a fold change in geometrical MFI values (WT vs *Slpi* ^-/-^). **(I)** Representative histograms of SLPI expression in blood monocytes isolated from WT (black line) and *Slpi* ^-/-^ mice (shaded histogram) injected with PBS or LPS for 24h. **(A, C, D, F, H)** Data represent 7 to 8 mice per experimental group pooled from three independent experiments Error bars show means ± SEM. **(A, C, D, F)** Control vs LPS or WT vs *Slpi* ^-/-^ * p<0.05, **p<0.01, ***p<0.001, ****p<0.0001 by one-way ANOVA, Tukey *post hoc* test. **(H)** WT Ly6C^hi^ monocytes vs WT Ly6C^low^ monocytes ****p<0.0001 by one-way ANOVA, Tukey *post hoc* test.

As LPS induces systemic inflammation, we compared inflammatory responses in circulation. LPS injection induced leukopenia, evidenced by a decrease in CD45^+^ cells in the blood (**Figure 6A**). Following detailed analysis of myeloid populations, we observed substantial changes within these cells. For instance, neutrophil and monocyte frequency were increased while eosinophils were decreased following LPS injection (**Figure 6B, C**). Interestingly, SLPI had an impact on blood leukocytes, as the frequency of monocytes was significantly decreased in *Slpi* ^-/-^ mice, while neutrophils and eosinophils were comparable (**Figure 6B, C**). When we analyzed monocyte population in detail, we observed that in PBS groups, there was one main CD11b^+^Ly6C^high^ population and in LPS there were two additional distinct populations: CD11b^+^Ly6C^high^MHC II^+^F4/80^+^ and CD11b^+^Ly6C^low^MHC II^-^F4/80^-^(**Figure 6D, E**). The frequency of Ly6C^high^ monocytes remained similar in PBS and LPS group in WT mice, however in *Slpi* ^-/-^ mice they were significantly decreased in LPS group (**Figure 6D**). Two monocyte populations, MHC II^+^F4/80^+^ and Ly6C^low^ were increased in LPS group, however in *Slpi* ^-/-^ mice these populations were circulating at lower levels (**Figure 6D**). Overall, this suggests that lack of SLPI influences various monocyte populations. Next, we assessed SLPI expression in blood leukocytes and detected significant changes in SLPI expression in myeloid populations. For instance, in PBS groups, SLPI was expressed by neutrophils, but following LPS treatment it was decreased (**Figure 6F, G**). On the other hand, Ly6C^+^ monocytes expressed similar levels of SLPI in PBS and LPS groups (**Figure 6H, I**). In addition, two monocyte populations which were recruited in LPS group were also SLPI^+^. Interestingly, MHC II^+^F4/80^+^ monocytes expressed low levels of SLPI, while Ly6C^low^ monocytes expressed high levels of SLPI (**Figure 6H, I**). Overall, these results show that SLPI expression is modulated during systemic inflammation in various leukocytes.

## Discussion

Macrophages consist of multiple and heterogeneous populations that can be found in every tissue of the body. They play various functions; therefore, regulation of their responses is critical for maintaining local homeostasis (59). SLPI is one of the proteinase inhibitors which has been investigated for its role in the regulation of inflammatory responses in many immune cells, including macrophages. However, initial research on SLPI did not take into account that tissue-resident macrophages are versatile and that this protease inhibitor might play distinct roles depending on the tissue context.

The interest in SLPI as an anti-inflammatory molecule stem from initial research which showed that SLPI is upregulated during LPS stimulation and inhibits TNF secretion (36). This report was followed by several others showing that SLPI interfere with LPS uptake (28) and inhibits NF-κB signaling pathway (15, 16). However, it is crucial to note that these reports relied on overexpression or addition of exogenous SLPI and cell lines. Furthermore, there were also studies showing that SLPI did not impact TNF production in macrophages (29, 37). In general, these various phenotypes can be due to different experimental conditions and specific assays, but we also speculate that SLPI might have pleiotropic effects on specific myeloid cell populations.

Here we show that primary macrophages of different tissue origin are not equal and they express *Slpi* mRNA and SLPI protein at various levels. Macrophages perform both universal and tissue-specific functions in their local microenvironment. Large peritoneal macrophages maintain local homeostasis, but also contribute pathogen defense and injury repair (38). Alveolar macrophages are responsible for alveolar homeostasis and surfactant degradation (60). Splenic macrophages which are enriched mostly by red pulp macrophages which are crucial for iron recycling (61). As these cells represent diverse lineages, local environments and functions it remains to be established what type of cues drive SLPI expression in each population. Moreover, as we focused in this study on peritoneal macrophages, which have the highest SLPI expression, it remains to be investigated whether SLPI plays similar or distinct roles in other tissue macrophages.

Furthermore, we confirmed that peritoneal macrophages express high levels of SLPI in steady state in contrast to BMDMs. Interestingly, culturing peritoneal macrophages without any stimulation induces SLPI secretion, which shows that these cells are natural producers of SLPI. These results prompted us to investigate the role of SLPI in peritoneal macrophages.

In resident peritoneal macrophages, SLPI did not regulate TNF and IL-6 secretion after LPS stimulation. On the other hand, in TGMs, SLPI expression decreased IL-6 production, while TNF was marginally affected. These results would suggest that SLPI regulates different signaling pathways in myeloid cells. Furthermore, we show that endogenous SLPI does not inhibit IL-1 release from resident peritoneal macrophages. However, we cannot rule out that SLPI can be involved in IL-1 activation at different pathways, as exogenous SLPI or its overexpression were shown to inhibit ATP-induced release of IL-1β (44), while we tested other NLRP3 inducers. Moreover, in recent years it became apparent that IL-1 family members are processed by intracellular and extracellular enzymes. For instance, pro-IL-1α which is active without any processing can be further activated by granzyme B and elastase (62, 63). On the other hand, pro-IL-1β is processed by intracellular caspase-1 to its active form, but then it can be degraded by neutrophil-derived proteases such as elastase and cathepsin G (63). As SLPI is an inhibitor of several neutrophil-derived proteases, we hypothesized that the lack of SLPI might change the activity of IL-1 *in vivo.* However, IL-1-dependent neutrophil recruitment to the site of alum injection was marginally affected by SLPI deficiency, which suggests that IL-1 activity is similar in WT and *Slpi^-/-^* mice. It is possible that other proteinase inhibitors might be sufficient to regulate IL-1 activity, thus making SLPI redundant in this model. Moreover, it is possible that SLPI plays a more profound role in other IL-1 family members activity such as IL-33, IL-36 or IL-37 which also undergo various enzymatic processing (63–67), but this hypothesis needs further investigation.

Having established that SLPI does not possess a broad anti-inflammatory property, we propose that SLPI plays a specific role in regulating specific macrophage responses. We show here, that SLPI is essential to limit MMP-9 secretion by resident and recruited peritoneal macrophages. Interestingly, in our experiments various TLR ligands induced MMP-9 upregulation in macrophages of different origin, which might also reflect their roles in environment sensing. It is possible that the increase of MMP-9 could be related to LPS-induced cell death, but this needs to be investigated in the future studies on the relationship between SLPI and MMP-9. Our results are in line with previous research on the role of SLPI in production of MMP-9 by monocytes and eosinophils (9, 68, 69). Specifically, it has been shown that addition of intact SLPI protein reduced MMP-9 secretion by activated monocytes, while cleaved SLPI did not (69). In accordance, it can be assumed that in WT peritoneal macrophages, endogenous SLPI restricts MMP-9 secretion, while *Slpi* ^-/-^ cells secrete higher levels of this mediator. However, it remains to be confirmed whether addition of exogenous SLPI would impact inflammatory response in peritoneal macrophages.

Furthermore, there is still a need to investigate whether increased secretion of MMP-9 by *Slpi* ^-/-^ macrophages would play a role in pathophysiological processes including tissue remodeling orchestrated by macrophages during disease. While we show that increased MMP-9 is more potent in zymography assay, this needs to be confirmed using *in vivo* models. From published studies, it is known that enhanced MMP-9 expression in macrophages induces its migration during inflammatory injury (70), mesenchymal transition in pancreatic cancer (71) or Wilms’ tumor metastasis (72). Thus, the role of SLPI in these models might be worth pursuing.

The link between SLPI and MMP-9 was reported in the context of cancer cell lines where it has been shown that SLPI can either limit MMP-9 secretion in ovarian cancer or promote MMP-9 in gastric cancer (73, 74). These results show that the relationship between these two mediators might be more complicated than a single mechanism. Moreover, it has been shown that engrafted tumor cell lines that express low levels of SLPI grow slower in *Slpi* ^-/-^ mice, while tumor cell lines with high SLPI expression form tumors similarly to WT mice. Furthermore, in chemically-induced lung cancer, *Slpi* ^-/-^ mice were protected from tumor formation (75). However, the mechanism of the protection in the absence of SLPI is not fully understood and it remains to be established whether MMP-9 plays a role in it *in vivo*. Overall, it is broadly accepted that SLPI plays an important role in tumorgenesis (25). Importantly, as cancer tissue is heterogeneous and consists of tumor and immune cells, there might be various sources and targets of SLPI with distinct functional outcomes.

Among other immune cells, neutrophils were also shown to express SLPI during homeostasis (6, 7). However, the knowledge of SLPI expression pattern within immune cells and during different diseases is just emerging. For example, mast cells express different levels of SLPI during pathophysiological settings, such as psoriasis when compared to healthy controls (8). We show that myeloid cells express SLPI at steady-state in neutrophils and eosinophils in blood and also in peritoneal macrophages. Interestingly, there are substantial changes in SLPI expression across myeloid cells during LPS-induced inflammation. Notably, one significant change is the downregulation of SLPI in neutrophils following LPS administration. As SLPI has been shown to be released as a part of neutrophil extracellular traps (76), it is possible that these differences in SLPI level are due to its release. It is also possible that endotoxin shock induces profound changes in granulopoiesis in bone marrow resulting in neutrophil efflux to blood with different expression of SLPI. However, it still remains to be explored how different levels of SLPI in neutrophils impact their functions.

We found that SLPI is significantly upregulated in blood monocytes following endotoxin shock. In homeostatic conditions, murine blood monocytes consist of two main populations, classical monocytes (CD11b^+^Ly6C^high^) and non-classical monocytes (CD11b^+^Ly6C^low^). Ly6C^low^ monocytes patrol and rapidly respond to inflammatory changes in the vasculature (77). In our *in vivo* model we were able to detect these two populations, and additionally we detected the inflammatory monocyte population which can be described as CD11b^+^Ly6C^high^MHC II^+^F4/80^+^. Interestingly, SLPI impacted the frequency of monocyte populations. Previously, it has been shown that exogenous SLPI protects monocytes from apoptosis (78) thus it is possible that lack of SLPI renders them more prone to cell death during LPS challenge *in vivo*. Furthermore, it remains to be elucidated how SLPI impacts various monocyte functions. We speculate that SLPI could regulate local environment either at vasculature or at the site of inflammation. For instance, it has been shown recently at the single cell RNA-Seq level that *Slpi* is expressed by monocytes associated with murine aortas and it is downregulated during atherosclerosis. It was speculated that changes in inhibitor expression might impact vascular remodeling (79). Interestingly, in our experiments lack of SLPI induced specific differences in inflammatory responses following LPS challenge *in vivo*. This is in contrast to a previous report, where it was shown that *Slpi^-/-^* mice were highly susceptible to LPS-induced endotoxin shock (17). However, it is possible that this difference is due to a different dose of LPS. In the previous study, 1 mg of LPS per mouse was used to induce inflammatory responses, while in the current study we used only 50 µg per mouse. Indeed, we show that higher LPS doses induce higher SLPI secretion during *in vitro* stimulation of macrophages. Thus, it is probable that during low dose of LPS *in vivo*, SLPI might not be critical for keeping the inflammatory response in check. For *in vivo* studies, it would be advantageous to perform similar experiments on myeloid-specific SLPI-deficient mice, which would show the specific role of myeloid cell-derived SLPI on its immunomodulatory functions rather than overall lack of this protein. However, our model still allowed us to discover new SLPI^+^ cell populations, which can be investigated in future.

Overall, our data highlight that SLPI performs various and distinct functions in immune cells, rather than having broad anti-inflammatory properties. Therefore, the specific roles of SLPI should be carefully evaluated in the context of different diseases, with a focus on its molecular and cellular targets.

## Data Availability

Publicly available datasets were analyzed in this study. These datasets can be found here: GEO: GSE179504 (https://www.ncbi.nlm.nih.gov/geo/query/acc.cgi?acc=GSE179504), SRA: PRJNA482293 (https://www.ncbi.nlm.nih.gov/sra/?term=PRJNA482293), iProX: IPX0001245000 and PXD021583 (https://www.iprox.cn/page/SCV017.html?query=IPX0001245000).
