# Correction: Endogenous SLPI contributes to the regulation of inflammatory responses in peritoneal macrophages by modulating MMP-9 production

**DOI:** 10.3389/fimmu.2025.1645241

**Published:** 2025-07-23

**Authors:** Mariia Tyshchenko, Natalia Pocałuń, Patrycja Kwiecińska, Joanna Cichy, Mieszko M. Wilk, Ewa Oleszycka

**Affiliations:** ^1^ Department of Immunology, Faculty of Biochemistry, Biophysics, and Biotechnology, Jagiellonian University, Kraków, Poland; ^2^ Doctoral School of Exact and Natural Sciences, Jagiellonian University, Kraków, Poland

**Keywords:** SLPI, proteinase inhibitor, LPS, septic shock, inflammation, monocytes, peritoneal macrophages, MMP-9 (matrix metalloproteinase-9)

There was a mistake in **Figure 3** as published. The final version of **Figure 3** which included additional panel of zymography (**Figure 3G**) was not included during post-acceptance proofs and pre-peer review version of **Figure 3** was published by mistake. The correct version of **Figure 3** appears below.

**Figure 3:**
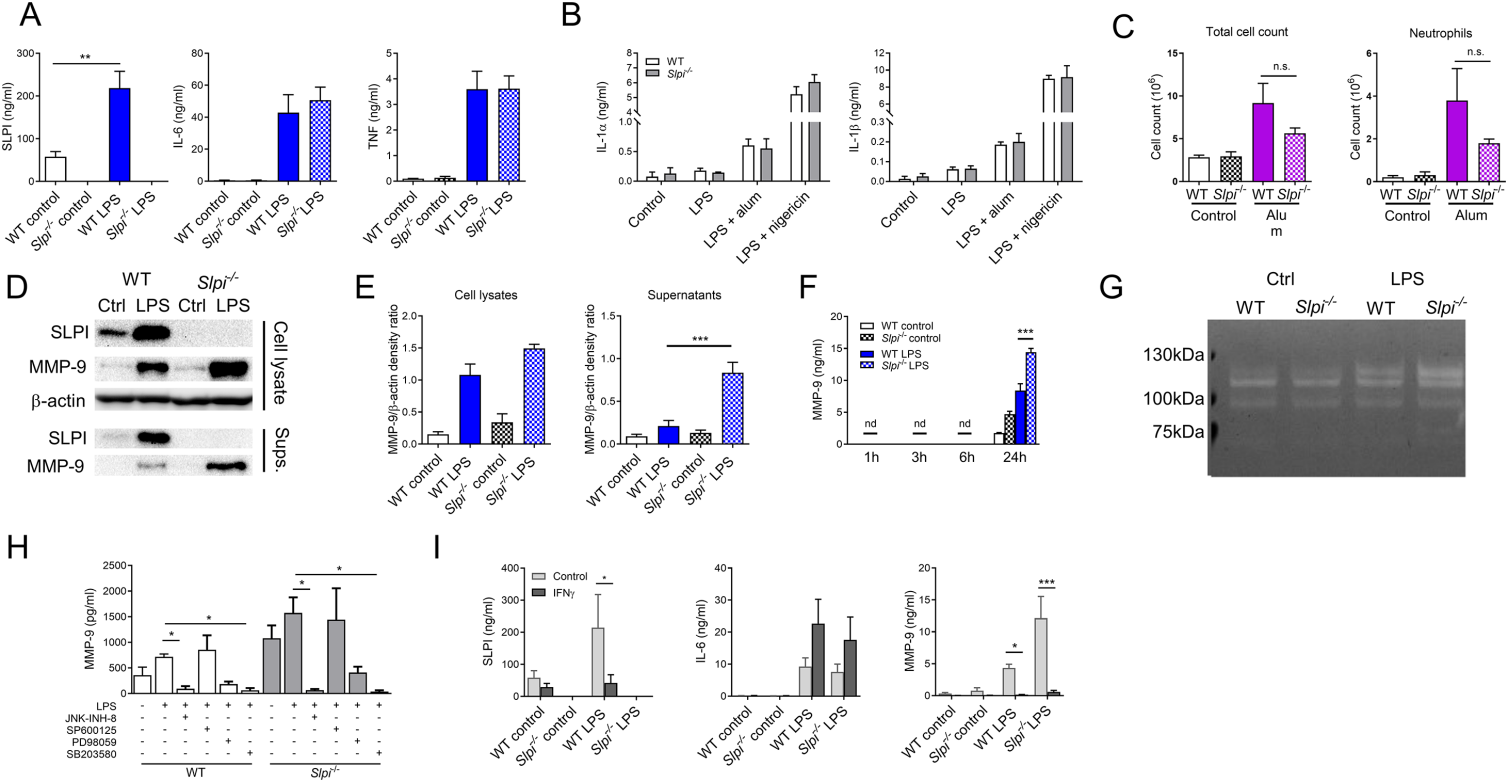
SLPI regulates MMP-9 secretion in resident peritoneal macrophages. (A) SLPI, IL-6 and TNF in supernatants of WT and Slpi^-/-^ resident peritoneal macrophages incubated with LPS (100 ng/ml) for 24h.WT control vs WT LPS ** p< 0.01 by one-way ANOVA, Tuckey post hoc test. (B) IL-1α and IL-1β in supernatants of WT and Slpi^-/-^ peritoneal macrophages incubated with LPS (100 ng/ml) for 3 h followed by addition of alum (100 µg/ml) or nigericin (10 µM). (C) Total cell and neutrophil count in WT and Slpi^-/-^ mice injected i.p. with PBS or 100 μg alum for 24h. (D) Representative immunoblots of MMP-9 and SLPI in supernatants and lysates of WT and Slpi^-/-^ peritoneal macrophages incubated with LPS (100 ng/ml) for 24h.Samples were resolved by SDS-PAGE and probed by Western blotting. β-actin was used as loading control. (E) Densitometry analysis of (D) WT LPS vs Slpi^-/-^ LPS ***p < 0.001 by one-way ANOVA, Tukey post hoc test. (F) MMP-9 in supernatants of WT and Slpi^-/-^ resident peritoneal macrophages incubated with LPS (100 ng/ml) for indicated times. WT LPS vs Slpi^-/-^ LPS ***p < 0.001 by one-way ANOVA, Tukey post hoc test. (G) Representative zymography of equal volume of supernatants obtained from WT and Slpi^-/-^ peritoneal macrophages incubated with LPS (100 ng/ml) for 24h.Representative of four separate experiments. (H) MMP-9 in supernatants of WT and Slpi^-/-^ resident peritoneal macrophages incubated with DMSO or inhibitors: JNK-INH-8 (10 μM), SP600125 (20 μM), PD98059 (20 μM) or SB203580 (10 μM) for 1h followed by LPS treatment (100 ng/ml) for 24h.LPS vs LPS + inhibitor *p<0.05 by one-way ANOVA, Tukey post hoc test. (I) SLPI, IL-6 and MMP-9 in supernatants of WT and Slpi^-/-^ resident peritoneal macrophages incubated with LPS (100 ng/ml) and IFN-γ (50 ng/ml) for 24h.Control vs IFN−γ *p<0.05, ***p<0.001 by multiple unpaired t-test. (A, B, F, H, I) Data are presented as the mean of three (A, B, F) or four (H, I) independent experiments. Error bars show means ± SEM. (C) Data represent 5 to 6 mice per experimental group pooled from two independent experiments. Error bars show means ± SEM.

