# Supplementary material for: Endogenous SLPI contributes to the regulation of inflammatory responses in peritoneal macrophages by modulating MMP-9 production
Source: Front Immunol. 2025 May 27;16:1563845. doi: 10.3389/fimmu.2025.1563845 (PMC12149117; doi:10.3389/fimmu.2025.1563845)
Supplement: Supplementary file 1 [file DataSheet1.pdf]

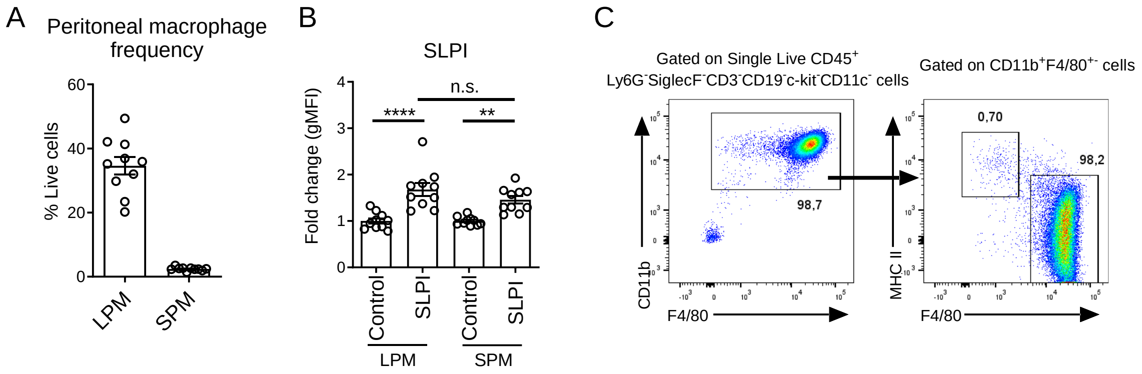

Supplementary Figure 1. Analysis of small and large peritoneal macrophages. (A) Frequency of LPMs (CD11b$^+$F4/80^{high}MHC II$^-$) and SPMs (CD11b$^+$F4/80^{low}MHC II$^+$) in naïve WT mice . Data pooled from n=10 (B) Fold change of geometrical MFI was compared between Control and anti-SLPI staining in macrophages. Data represent 10 mice per experimental group pooled from two independent experiments. Control vs anti-SLPI ** $p < 0.01$, **** $p < 0.0001$ by one-way ANOVA, Tukey post hoc test. (C) Representative flow cytometry analysis of MACS-sorted LPMs (CD11b$^+$F4/80^{high}MHC II$^-$) and SPMs (CD11b$^+$F4/80^{low}MHC II$^+$) in naïve WT mice.

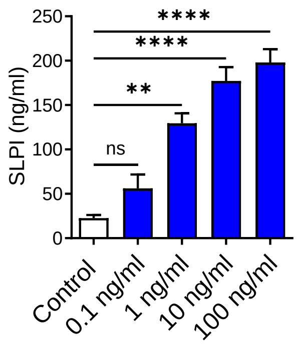

Supplementary Figure 2. The impact of LPS dose on SLPI secretion by peritoneal macrophages. SLPI in supernatants of WT resident peritoneal macrophages incubated with LPS (0.1-100 ng/ml) for 24h. Data are presented as the mean of three independent experiments. Error bars show means ± SEM. Control vs LPS ** $p < 0.01$, ****$p<0.0001$ by one-way ANOVA, Tukey post hoc test.

Supplementary Figure 3. Uncropped blots and zymography relating to Figure 3

MMP-9 (left – cell lysates, right - supernatants)

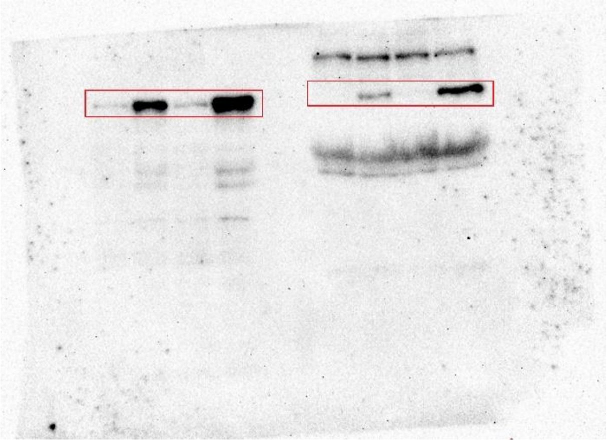

Protein ladder for MMP-9 blot

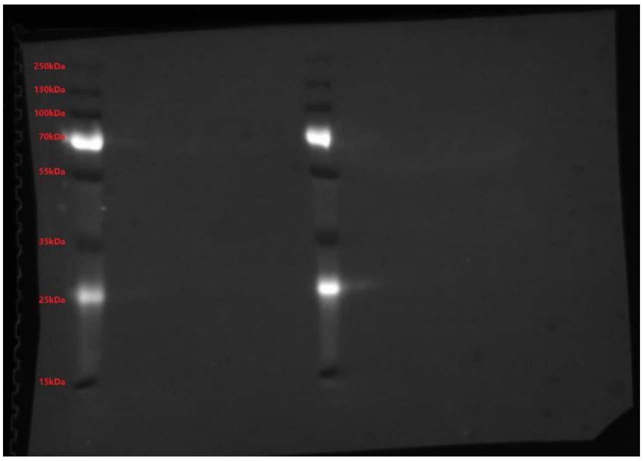

SLPI (left -cell lysates)

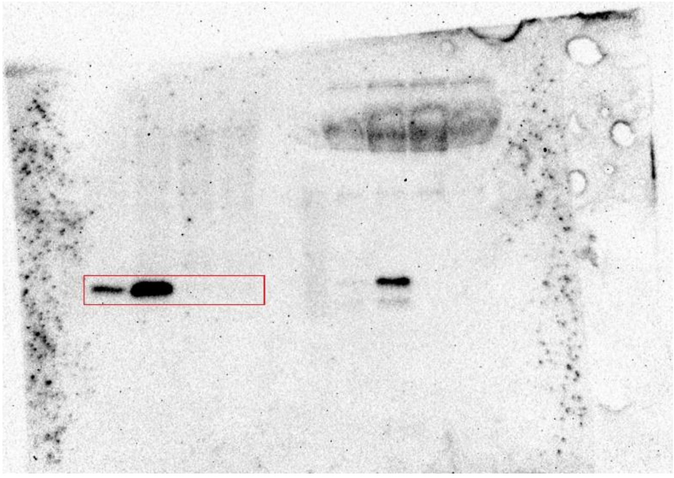

SLPI (right – supernatants, longer exposure, manually cropped from blot on the left)

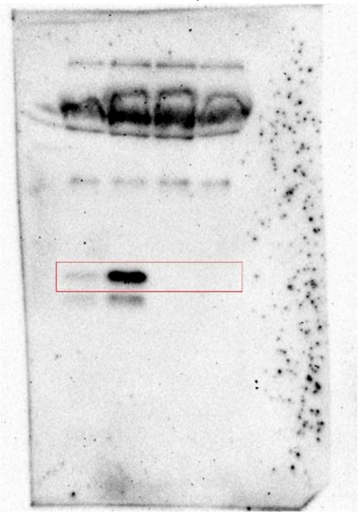

Protein ladder for SLPI blot

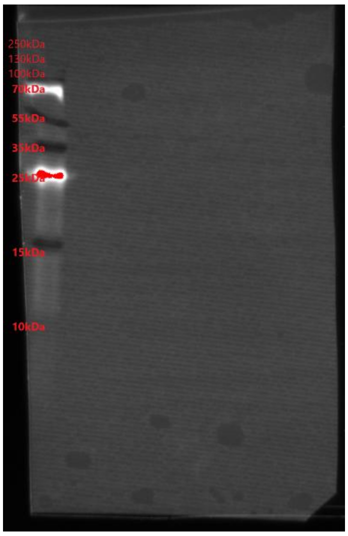

β-actin (developed on the same membrane as MMP-9 blot, manually cropped for cell lysates)

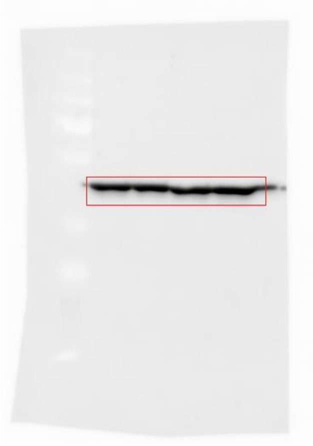

Zymography

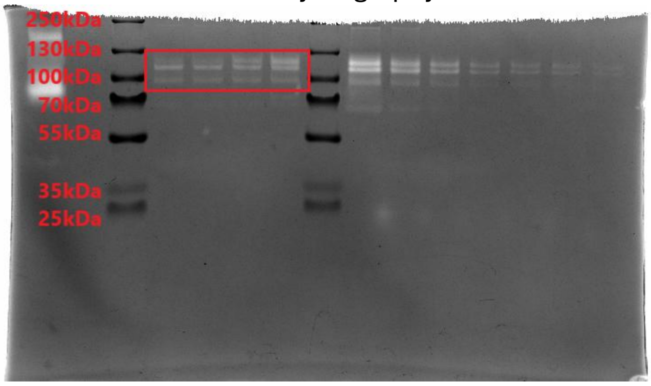

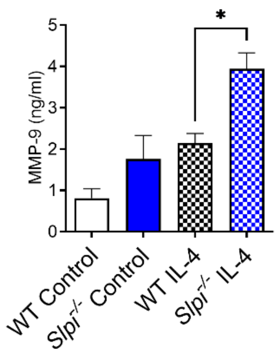

Supplementary Figure 4. The impact of IL-4 on MMP-9 secretion by peritoneal macrophages. SLPI in supernatants of WT resident peritoneal macrophages incubated with IL-4 (40 ng/ml) for 24h. Data are presented as the mean of three independent experiments. Error bars show means ± SEM. Control vs LPS * $p < 0.05$ by one-way ANOVA, Tuckey post hoc test.
